# Temporal Pattern of the Emergence of a Mutant Virus Escaping Cross-Immunity and Stochastic Extinction Within a Host

**DOI:** 10.1007/s11538-023-01184-x

**Published:** 2023-07-28

**Authors:** Rena Hayashi, Yoh Iwasa

**Affiliations:** Department of Biology, Faculty of Science, Kyushu University, 744 Motooka, Nishi-ku, Fukuoka, 819-0395 Japan

**Keywords:** Viral dynamics, Time-dependent branching processes, Cross-immunity, Escape mutants per patient

## Abstract

A high mutation rate of the RNA virus results in the emergence of novel mutants that may escape the immunity activated by the original (wild-type) strain. However, many of them go extinct because of the stochasticity due to the small initial number of infected cells. In a previous paper, we studied the probability of escaping stochastic extinction when the novel mutant has a faster rate of infection and when it is resistant to a drug that suppresses the wild-type virus. In this study, we examine the effect of escaping the immune reaction of the host. Based on a continuous-time branching process with time-dependent rates, we conclude the chance for a mutant strain to be established $p\left(t\right)$ decreases with time $t$ since the wild-type infection when the mutant is produced. The number of novel mutants that can escape extinction risk has a peak soon after the wild-type infection. The number of novel escape mutations produced per patient in the early phase of host infection is small both for very strong and very weak immune responses, and it attains its maximum value when immune activity is of an intermediate strength.

## Introduction

Within the host body, the virus infects target cells, increases the number of copies using host cell machinery, and is transmitted to the uninfected target cells. The replication error of their genome may produce mutants that are more efficient in some key steps of the viral life cycle. The mutants with a faster rate of proliferation or a weaker suppression by immunity than the original strain (or wild-type virus) would increase in number and replace the wild-type strain within the host. However, because these mutants start from a small number of infected cells (often a single infected cell), they suffer strong stochasticity, driving them to extinction. As a result, a considerable fraction of these advantageous mutants go extinct because of the stochasticity caused by the small number of infected cells. While the number of infected cells is small, stochasticity dominates the dynamics of the mutant strain. Once their number becomes sufficiently large, their deterministic increasing ability dominates stochasticity, and extinction becomes unlikely.

In a previous study (Hayashi et al. (2022)), we calculated the fraction of mutants that can escape stochastic extinction, based on a continuous-time branching process with a time-dependent growth rate. The chance for a mutant strain to be established decreases with the time since the infection of the wild-type strain, and it shows damped oscillation before convergence to the low stationary value. We also calculated the probability of escaping stochastic extinction for a drug-resistant mutant when a patient received an antiviral drug that suppressed the wild-type strain. Combining the rate of mutant production from the wild-type strain and the chance of escaping stochastic extinction, the number of emerging drug-resistant mutations may have two peaks: one soon after the infection of the wild-type strain and the second at the start of antiviral drug administration (Hayashi et al. 2022). However, the previous study did not consider the effect of the immune system, which is activated by the abundant wild-type strain and may partially suppress the proliferation of mutant strains.

In the present paper as a sequel to Hayashi et al. (2022), we study the probability of escape from initial stochastic extinction owing to the small number of infected cells when the mutant and the wild-type strains differ in their ability to stimulate immune responses. We evaluate how the fraction of mutants that survive stochastic extinction changes with the time at which they are produced. To illustrate, we consider the following scenario: before the viral infection, the number of target cells in the host was maintained at equilibrium. At a certain time, the host becomes infected with a viral strain that proliferates rapidly, reducing the number of susceptible target cells and activating the immune system. The proliferation of the virus is suppressed by the shortage of susceptible target cells and by the immune activity, leading to a stationary abundance of infected and target cells. Mutant strains may be produced owing to mistakes in the genomic replication of the original viral strain (or wild-type virus). Some of these are advantageous because they proliferate faster or evade immune surveillance more effectively than the wild-type strain. However, they suffer a high risk of stochastic extinction owing to the small number of infected cells.

**Fig. 1:**
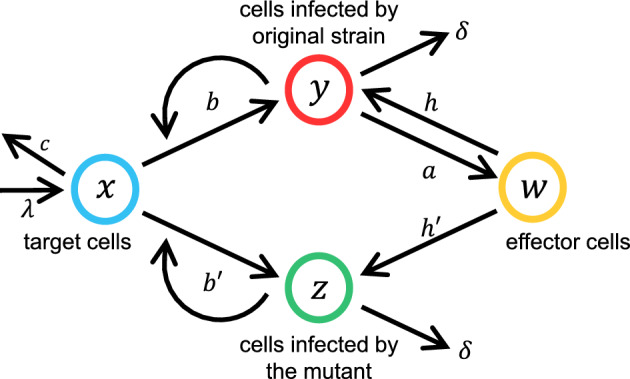
Schematic of the model. The four variables indicated in circles are the number of target cells $x$, the number of cells infected by the wild-type strain $y$, those infected by the mutant strain $z$, and the number of killer T cells $w$. We consider the situation in which $z$ is small and requires consideration of stochasticity in the dynamics of $z$. In contrast, because three other variables are much larger than unity, we can adopt deterministic dynamics for them. We focus on the time frame in which $z$ does not affect the dynamics of $x$, $y$, and $w$, rather the dynamics of $z$ are strongly affected by $x$ and $w$, both of which depend on time

We apply the mathematical analysis adopted in Hayashi et al. (2022) to the case with immune reaction of the host. To make the analysis of branching process applicable, we consider the escape (establishment) probability of a mutant virus under the following setting (see Fig. 1 for the scheme of the model): The number of cells infected by the wild-type strain is sufficiently large and their dynamics can be handled by a differential equation, deterministic dynamics. In contrast, a mutant strain starts from a single infected cell and has a high chance of stochastic extinction. Because the number of cells infected by the mutant virus is small, the reduction in susceptible target cells due to infection by the mutant strain can be neglected in the dynamics of target cells. In contrast, the proliferation rate of the mutant critically depends on both the abundance of susceptible target cells and the intensity of the immune reaction.

Under this setting, the fate of a lineage starting from a single cell infected by a mutant strain is independent of the presence or the prevalence of other mutant strains. The independence between lineages is the key assumption that allows us to adopt branching process calculation (see (Hayashi et al. 2022)). The number of mutant-infected cells follows a continuous-time branching process, where the mean rates of proliferation and mortality change with time following the dynamics of the target cells and immune activity. We derive a differential equation for the escape (establishment) probability of a mutant $p\left(t\right)$ as a function of the time $t$ at which the mutant is produced. We obtain an explicit mathematical formula for $p\left(t\right)$. The escape probability of a mutant $p\left(t\right)$ exhibits characteristic temporal patterns: it starts at a high value, then decreases with time, and converges to a stationary level after damped oscillation. We examine the parameter dependence of the temporal pattern of escape probability and the number of novel mutant viruses produced in a single patient.

## Model

Let $x$, $y$, and $w$ be the number of susceptible target cells, number of cells infected by the wild-type virus, and number of immune effector cells (e.g., activated killer T cells), respectively (see Fig. 1). We consider the following dynamics:

1a $$\frac{dx}{dt}=\lambda -cx-bxy$$

1b $$\frac{dy}{dt}=bxy-hwy-\delta y$$

1c $$\frac{dw}{dt}=ay-dw$$

Equation (1a) indicates the dynamics of susceptible target cells. The first term on the right-hand side indicates the supply of target cells at rate $\lambda$ and the second term indicates the loss of target cells with a turnover rate of $c$. Without viral infection, the target cells have a mean longevity of $1/c$. The third term indicates the rate of infection of the target cells by the virus, which is proportional to the number of infected cells $y$.

Equation (1b) indicates the dynamics of the number of cells infected by the virus. The first term on the right-hand side is the total rate of infection (transition from susceptible cells to infected cells), which is the same as the third term in Eq. (1a). The second term indicates the removal of infected cells by the action of cytotoxic T lymphocytes (i.e., killer T cells), and the third term indicates the mortality of infected cells by other processes. The instantaneous mortality of the infected cells is $hw+\delta$, which increases linearly with the number of active killer T cells $w$.

Equation (1c) gives the dynamics of the number of active killer T cells. They are formed by the activation of naïve killer T cells through the action of antigen-presenting cells, and subsequent proliferation by cell division of a finite number of times. The rate of production of active killer T cells is proportional to the abundance of viral antigens, which increases with the number of infected cells $y$. The proportionality coefficient is $a$, as indicated in the first term on the right-hand side. This functional form was called "linear immune response" by Nowak and May (2000) and was adopted in Iwasa et al. (2021). In the following analysis, we adopt this simplest form, although more complex functional forms of immune activation have also been studied (Boer et al. 2001). The second term on the right-hand side is the loss of activated killer T cells owing to mortality at rate $d$. The mean lifetime of killer T cells is $1/d$. If we neglect the function of killer T cells (either $h=0$ or $w=0$), the model is the same as that used in our previous study (Hayashi et al. 2022).

**Fig. 2:**
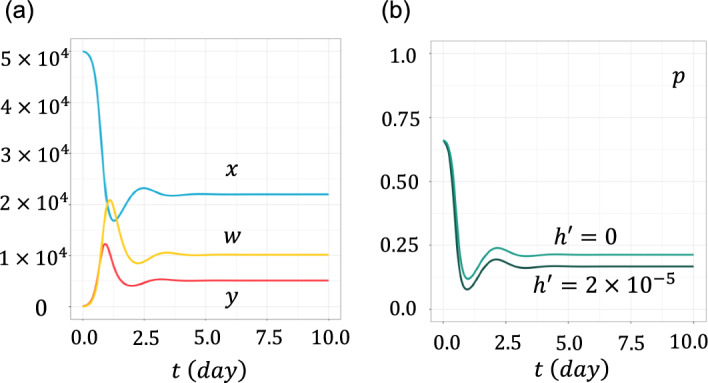
Time dependent solutions. **a** Numbers of susceptible target cells $$x\left(t\right)$$, infected target cells $$y\left(t\right)$$, and killer T cells $$w\left(t\right)$$. The horizontal axis represents time $$t$$ at which the mutant is produced. **b** Escape probability of a mutant virus starting from a single infected cell at time $$t$$, $$p\left(t\right)$$. Two curves are for different $$h^{{\prime } }$$: $$h^{{\prime } }$$$$=0$$, and $$h^{{\prime } } = 2 \times 10^{ - 5} ( < h)$$. Other parameters were: $$a=10$$, $$b=2.5\times {10}^{-4}$$, $$c=1$$, $$d=5$$, $$\delta =5$$, $$\lambda =5\times {10}^{4}$$, $$h=5\times {10}^{-5}$$, $$b^{{\prime } } = 3 \times 10^{ - 4}$$, and $$\varepsilon =100$$

Because $x$, $y$ and $w$ are large numbers, we can assume them as the deterministic differential equations given in Eq. (1). Figure 2 illustrates the case with the following scenario: Initially, there was no virus ($y=0$). The abundance of the target cells was calculated using Eq. (1a) with $y=0$, and converged to $x=\lambda /c$. The wild-type virus then invaded with a very low initial abundance. For simplicity of argument, we set the infection time to $t=0$. We denoted the initial abundance of the virus as $y\left(0\right)=\varepsilon$, where $\varepsilon >0$ was positive but much smaller than $\lambda /c$. Viral prevalence $y$ measured in terms of the number of infected cells increased and then decreased because the target cells decreased and killer T cells $w$ increased in number. Finally, it converged to a positive value $y\left(\infty \right)$. At equilibrium, the abundance of susceptible target cells $x\left(\infty \right)$ and the intensity of immune reactions $w\left(\infty \right)$ were positive.

The mutants are produced from the original (wild-type) strain at a very low rate. Because a newly produced mutant strain starts from a single infected cell, it has a high risk of extinction even if it has a positive mean growth rate. The escape probability of a mutant strain depends on the time $t$ at which the mutant is produced ($t>0$). The mean per capita rate of increase of the mutant infected cells decreases with time because the abundance of target cells $x\left(t\right)$ declines and the immune reaction $w\left(t\right)$ increases as the wild-type strain proliferates. Hence, mutants produced earlier have a higher chance of escape than those produced later.

The mutant has an infection rate $b^{{\prime } }$ and the effectiveness of immune reaction $h^{{\prime } }$ (i.e. efficiency of cytotoxic T lymphocytes), which may differ from the corresponding rates for the wild-type strain ($b$ and $h$, respectively). Because the mutant is produced from the wild-type viral strain, we consider the situation in which the wild-type virus has a positive abundance. This precludes the case of $\lambda /c\le \delta /b$, for which the wild-type strain cannot be maintained in the host body (Appendix A). Hence, we assume $\lambda /c>\delta /b$ in the following.

To simplify the mathematical analysis, we assume that the stochasticity caused by the small number of infected cells is important for the mutant strain, but not for the wild-type strain. Even if the initial abundance of the wild-type strain $y\left(0\right)=\varepsilon$ is much smaller than the abundance of the susceptible target cells, it can be sufficiently larger than 1. The dynamics of $x$, $y$, and $w$ are then described by the deterministic dynamics given by Eq. (1).

Let $z$ be the number of cells infected with the mutant strain. It is produced by rare mutations at the time of replication of the wild-type strain. We assume that $z$ starts from 1. The dynamics of $z$ can be expressed as:

2 $$\frac{dz}{dt}={b}^{{{\prime }}}xz-{h}^{{{\prime }}}wz-\delta z+[\mathrm{stochastic fluctuation}]$$

where the last term on the right-hand side indicates stochastic fluctuation with a mean of zero. If the infection rate of the mutant strain is higher than that of the wild-type strain ($b^{{\prime } } > b$), the immune suppression on the mutant is weaker than that on the wild-type strain ($h^{{\prime } } < h$), or both, $z$ increases on average over time (otherwise the mutant becomes extinct with probability one). However, the stochasticity caused by the small number of cells results in the extinction of mutant with a considerable probability. Once the abundance of the number of infected cells becomes sufficiently large, the effect of stochasticity is weaker than the deterministic rate of increase, and thereafter the cell number $z$ continues to increase exponentially.

Because we focus on the situation in which the number of target cells infected by the mutant strain is much smaller than that of cells infected by the wild-type virus, we neglect the effect of the mutant strain on the abundance of susceptible target cells. Hence, Eq. (1a) does not contain a term for $z$.

## Escape Probability of a Mutant

We represent the dynamics of the number of infected cells by stochastic process in which $z$ is an integer ($z=0, 1, 2, 3,..$.). For simplicity of mathematical analysis, we first consider the viral transmission by cell-to-cell contact, in which an infected cell encounters a susceptible cell and infects it at a rate proportional to $x\left(t\right)$. The transmission via cell-to-cell contact was proved in the experiment with HIV (Iwami et al. 2015). Alternatively, viruses may proliferate to produce free viral particles that infect susceptible cells, in which a single infected cell ruptures, resulting in a number of newly infected cells with a Poisson distribution. The latter model for viral proliferation will be discussed later.

Equation (2) indicates that $z$ increases with infection $b^{{\prime } } x\left( t \right)z$ and decreases with mortality $\left( {h^{{\prime } } w\left( t \right) + \delta } \right)z$, which are both proportional to $z$, suggesting that these events occur independently between infected cells. Thus, we can handle the stochastic change in $z$ by focusing on the events experienced by a single infected cell. Suppose that a cell infected by a viral strain infects many other cells by transmission of the virus. Then, we regard these cells as "descendants" of the wild-type cell in this study. This usage of "lineage terminology" is useful for explaining the model, in which we treat the virus as a focal agent, and cells as their habitat (Hayashi et al. 2022).

We consider a single cell infected by the mutant strain at time $t$ and ask the provability of the lineage not to extinct within a finite time. This can be calculated by considering $p\left(t\right)$ as the probability that the lineage starting from the focal cell does not go extinct by time $T$, and examining the limit when $T$ is infinitely large. To derive the differential equation for $p\left(t\right)$, we decompose it according to the alternative events occurring in a short interval from $t$ to $t+\Delta t$, which are given as follows.

(i) The cell encounters with an uninfected target cell and virus is transmitted (generating two "offspring" cells) with a probability $b^{^{\prime }}x\left( t \right){\Delta }t$.
(ii) The cell dies with a probability $\left( {h^{{\prime } } w\left( t \right) + \delta } \right){\Delta }t$.
(iii) The cell remains unchanged with probability $$1 - b^{{\prime } } x\left( t \right){\Delta }t - \left( {h^{{\prime } } w\left( t \right) + \delta } \right)$$$$\Delta t$$

In these three events, the number of infected cells are (i) two, (ii) zero, and (iii) one, respectively. Then, we have the following equation:

3 $$p\left( t \right) = b^{{\prime } } \left( {t } \right) x\left( t \right)\Delta t{p}_{2}\left(t+\Delta t\right) +\delta\Delta t\cdot0 + \left(1 - b^{{\prime } } x\left( t \right){\Delta }t - \left( {h^{{\prime } } w\left( t \right) + \delta } \right)\Delta t\right)p\left( t +\Delta t\right) + o\left( {{\Delta }t} \right)$$

where ${p}_{2}\left(t+\Delta t\right)$ is the probability that the lineage starting from two initial cells at time $t+\Delta t$ does not go extinct by time $T$. This explains the first term on the right-hand side of Eq. (3). If the cell dies by $t+\Delta t$, the probability for the focal lineage to escape by time $T$ is zero, which explains the second term on the right-hand side of Eq. (3). The third term is the case when the infected cell remains unchanged.

The behavior of the lineages starting from different cells is assumed independent of each other, which is a key premise of branching process. We have ${p}_{2}\left(t\right)=1-{\left(1-p\left(t\right)\right)}^{2}$ because the extinction of descendants starting from two cells is equivalent to the extinction of both lineages starting from one cell. The last term in Eq. (3), $o\left(\Delta t\right)$, indicates a small quantity of higher order than $\Delta t$, which satisfies $o\left(\Delta t\right)/\Delta t\to 0$ when $\Delta t\to 0$. Hence, as $\Delta t\to 0$, Eq. (3) becomes the following:

4 $$-\frac{dp}{dt}=b^{\prime }x\left(t\right)p\left(t\right)\left[1-p\left(t\right)\right]-\left(h^{\prime }w\left(t\right)+\delta \right)p\left(t\right)$$

For detailed explanation of the derivation of Eq. (4), see Appendix B.

We can calculate $p\left(t\right)$ by integrating Eq. (4) with respect to $t$ using the terminal condition $p\left(T\right)=1$. Because we here ask whether or not a newly produced mutant virus can be established in the target cell population, we consider the behavior at the limit $T\to \infty$. As explained in Appendix B, Eq. (4) can be solved, as follows:

5 $$p\left(t\right)=\frac{1}{{\int }_{t}^{\infty }b^{\prime }x\left(s\right)exp\left[-{\int }_{t}^{s}\left(b^{\prime }x\left(t^{\prime }\right)-\left(h^{\prime }w\left(t^{\prime }\right)+\delta \right)\right)dt^{\prime }\right]ds}$$

We first calculate the target cell abundance and immune activities $\left\{x\left(t\right)\mathrm{ and} w\left(t\right),\mathrm{ for }t>0\right\}$ using Eq. (1). Next, we obtain the escape probability from Eq. (5).

### Trajectories of Escape Probability ${\varvec{p}}\left({\varvec{t}}\right)$

The vertical axis indicates $x\left(t\right)$, $y\left(t\right)$, and $w\left(t\right)$ in Fig. 2a, and the escape chance of the mutant $p\left(t\right)$ in Fig. 2b, which is calculated from Eq. (5). The horizontal axis indicates time $t$. We also performed direct simulations of the stochastic process. An explanation of this method can be found in our previous paper (Hayashi et al. 2022). These results are consistent with the predictions of the mathematical formula.

The escape probability of a mutant $p\left(t\right)$ starts from the highest value $p\left(0\right)$ because the wild-type viral type $y\left(t\right)$, a competitor for the mutant strain sharing the common resources $x\left(t\right)$, is initially small. As competitor abundance increases, the probability for the focal mutant to establish decreases. Figure 2b illustrates $p\left(t\right)$ in two cases that differ in the value of $h^{{\prime } }$, but in which the two stains have the same rate of infection of the target cells ($b^{{\prime } } = b$). When the mutant escapes the immune attack developed by the wild-type strain ($h^{{\prime } } = 0$), there is no cross-immunity. The mutant has a positive escape probability because it has an advantage over its competitor in that it is not attacked by immunity. When the mutant suffered an immune attack with an intensity lower than that of the wild-type strain ($0 < h^{{\prime } } < h$), cross-immunity exists but is weaker than the immunity to the wild-type type. Therefore, $p\left( t \right)$ is positive but is smaller than that in the case where $h^{{\prime } } = 0$.

## Number of Mutations that Escape Stochastic Extinction

The production rate of novel mutants is proportional to the number of infection events per unit time, $bx\left(t\right)y\left(t\right)$. Because the fraction of novel mutations that can escape the initial high risk of stochastic extinction is $p\left(t\right)$, the rate of production of novel mutants that will eventually be established is proportional to their product $f\left(t\right)=bx\left(t\right)y\left(t\right)p\left(t\right)$, although it needs to be multiplied by the mutation rate. We examine this quantity $f\left(t\right)$ as an indicator of the emergence rate of novel mutants as a function of time $t$.

**Fig. 3:**
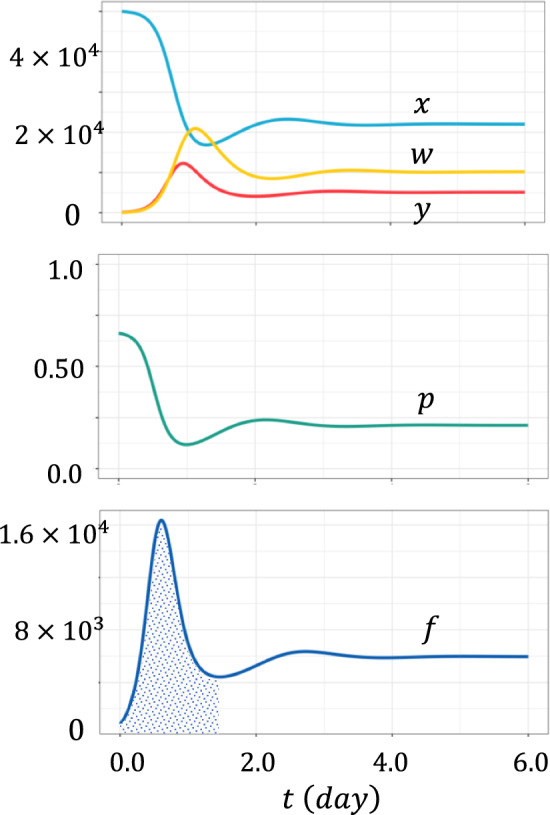
An indicator for the rate of novel mutant production $f\left(t\right)$. Horizontal axis is the time at which mutants are produced. We can see that the curve for $f\left(t\right)$ has a sharp peak near the onset of the infection. We numerically calculated the area under the curve between 0 and ${t}_{1}$, the latter being the time for the first local minimum of function $f\left(t\right)$. We call the size of the shaded area, ${F}_{M}={\int }_{0}^{{t}_{1}}f\left(t\right)dt$, "potential of novel mutant production in the early phase of infection." Standard set of the parameters were: $a=10$, $b=2.5\times {10}^{-4}$, $c=1$, $d=5$, $\delta =5$, $\lambda =5\times {10}^{4}$, $h=5\times {10}^{-5}$, $b^{{\prime } } = 3 \times 10^{ - 4}$, $h^{{\prime } } = 2 \times 10^{ - 5}$, and $\varepsilon =100$

An example is shown in Fig. 3. The top part indicates $x\left(t\right)$, $y\left(t\right)$, and $w\left(t\right)$, the middle part illustrates $p\left(t\right)$, the escape probability. The horizontal axis represents time $t$. The bottom part of the figure illustrates $f\left(t\right)$, an indicator of the production rate of mutations surviving stochastic extinction, which has a sharp peak shortly after the start of infection. Let ${\tau }_{f}$ be the time interval between the start of infection with the wild-type strain and the time when quantity $f\left(t\right)$ attains its maximum value. The height of the peak is $f\left({\tau }_{f}\right)$. In Appendix C, we show the sensitivities of ${\tau }_{f}$ and $f\left({\tau }_{f}\right)$ on the parameters in the model in terms of elasticity.

Furthermore, we consider the expected number of novel mutants that escape stochastic extinction per patient. The integral of $f\left(t\right)=bx\left(t\right)y\left(t\right)p\left(t\right)$ over the peak gives the expected number of mutants produced that did not go extinct within the host. Let ${t}_{1}$ be the time at the local minimum of $f\left(t\right)$ just after the first peak. ${F}_{M}={\int }_{0}^{{t}_{1}}f\left(t\right)dt$ is the potential for novel mutations that escape the stochastic extinction in the early phase of infection. ${F}_{M}$ is proportional to the expected number of novel escape mutations per patient.

**Fig. 4:**
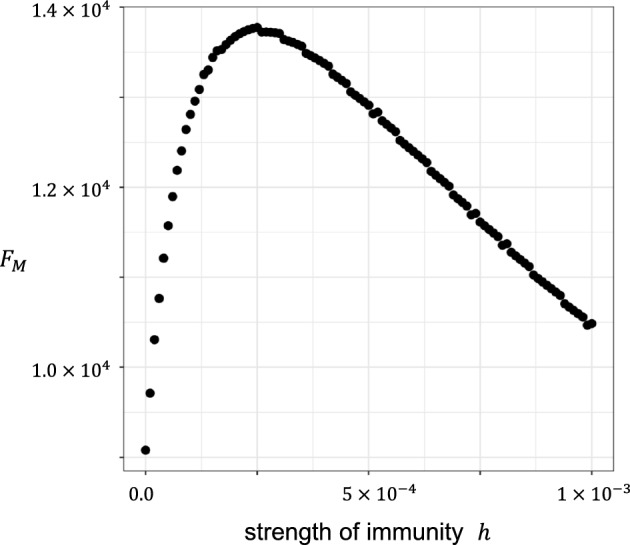
Potential of novel mutant production in the early phase of infection ${F}_{M}$. The horizontal axis represents the strength of immune reaction $h$, shown in the logarithmic scale. We fixed the ratio $h^{{\prime } } /h$ $(=0.4)$, and hence a large $h$ implies a large $h^{{\prime } }$. Sensitivity analysis showed that ${F}_{M}={\int }_{0}^{{t}_{1}}f\left(t\right)dt$, the potential of novel mutant production in the initial phase, is small both for extremely small $h$ and extremely large values of $h$; however, it achieves its maximum at an intermediate rate. Other parameters are the same as in Fig. 3

Figure 4 illustrates ${F}_{M}$. The horizontal axis is the magnitude of immune pressure $h$. Because we fixed the ratio $h^{{\prime } } /h$, a larger $h$ results in a larger $h^{{\prime } }$. ${F}_{M}$ is small under both strong and weak immune pressures. ${F}_{M}$ attains a maximum at an intermediate magnitude of immune pressure.

## Parameter Dependence

In the current paper and a twin paper (Hayashi et al. 2022), we showed the importance of stochastic extinction of mutant viral strains when they appear in a body of the host. The main aim of these papers is to search for the general property of the system. Numerical examples are simply to illustrate the possibility of the behavior of the model. Research activity of fitting the model to particular situations should be done separately.

Herein, we report the parameter dependence of the model's behavior known from introducing various approximations. Below, we describe the results briefly and the analyses are explained in Appendixes.

### Target Cell Abundance ${\varvec{x}}\left({\varvec{t}}\right)$

**Fig. 5:**
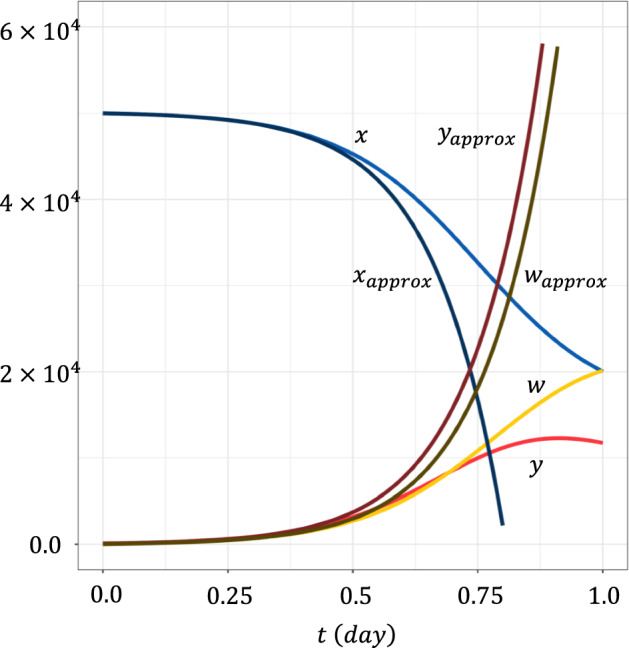
Trajectories of the approximate formulas for small $t$ (Eq. (D.1) in Appendix D). $x\left(t\right)$, $y\left(t\right)$, and $z\left(t\right)$ were accurate for the time close to the infection of the virus. Parameters were the same as in Fig. 3

The target cell abundance $x\left(t\right)$ starts from $x\left(0\right)=\lambda /c$ and decreases with time (Figs. 2, 3 and 5). The rate of decline is initially slow but becomes increasingly fast, because the abundance of virus infected cells starts from a small value and increases exponentially with time.

To characterize the time until $x\left(t\right)$ to start declining, we consider ${\tau }_{x}$, defined as the time for $x\left(t\right)$ to reduce by 20% of the initial level: $x\left({\tau }_{x}\right)=0.8x\left(0\right)$. We numerically derived ${\tau }_{x}$ for different sets of parameters and performed a sensitivity analysis, as explained in Appendix C.

As described in Appendix D, we derive the approximate formulas assuming that $t$ is small. We define $\widehat{x}\left(t\right)=x\left(t\right)-\lambda /c$. Then $\widehat{x}\left(t\right)$, $y\left(t\right)$, and $z\left(t\right)$ are of order $\varepsilon$ for a small $t$. We derive the linear dynamics for these three variables by neglecting the terms of the higher order with respect to $\varepsilon$ and solving them explicitly. All three include a dominant term that grows in proportion to $\varepsilon \cdot exp\left[\eta t\right]$ where $\eta =b\lambda /c-\delta$. For example, the abundance of the target cells is given by ${x}_{approx}\left(t\right)=\left(\lambda /c\right)\left[1-\left(\varepsilon b/\left(\eta +c\right)\right) \cdot \right.\left.exp\left[\eta t\right]\right]$. As shown in Fig. 5, we plotted this approximation along with the exact solution using Eq. (1). The approximation was accurate for small $t$; however, the deviation from the exact solution increased with time. The approximate formula for $x\left(t\right)$ provides an explicit solution for the time taken for the 20% decline in $x\left(t\right)$ as follows: ${\tau }_{x}=\frac{1}{\eta }log\left(\frac{0.2\left(\eta +c\right)}{\varepsilon b}\right)$ (see Appendix D). This formula underestimates the exact value of ${\tau }_{x}$ but is useful for determining parameter dependence.

After transient behavior including damped oscillation, $x\left(t\right)$ converges to a stationary value, which can be calculated as the value at the stable positive equilibrium of Eq. (1). See Appendix A for details.

### Escape Probability $p\left(t\right)$

Next, we consider $p\left(t\right)$, the probability for a mutant to be established depending on the time $t$ at which the mutant is produced.

#### Initial Value $p\left(0\right)$

**Fig. 6:**
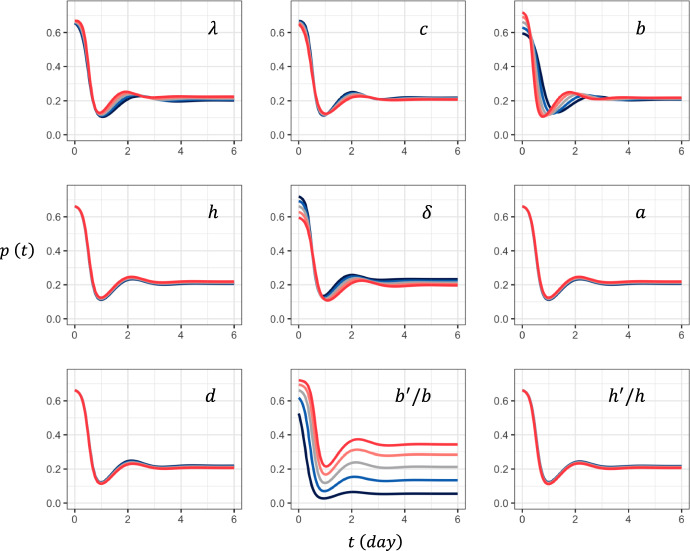
Parameter dependence of function $p\left(t\right)$. We set the standard parameter set, and changed parameters one by one by multiplying 1.2, 1.1, 1.0, $1/1.1=0.9091$, and $1/1.2=0.8333$, shown as curves in red, orange, gray, blue, and black, respectively. Parameters are the same as in Fig. 3

The escape probability is the highest just after the onset of infection by the wild-type strain ($t=0$), and it declines with time because the target cells are consumed and the immune reactions become stronger owing to the increase in the wild-type strain. In Fig. 6, the different parts illustrate the changes in curve $p\left(t\right)$ when one of the nine parameters is modified. In Appendix C, we numerically performed a sensitivity analysis of $p\left(0\right)$. The results are summarized as follows: $p\left(0\right)$ increases with $b^{{\prime } } /b$, $\lambda$, and $b$; it decreases with $c$ and $\delta$; however, it is independent of $h^{{\prime } } /h$, $h$, $a$, or $d$.

The lineage starting from a mutant produced at time $t$ experiences temporal changes in the availability of target cells $x\left(t\right)$ and in the intensity of the immune system $w\left(t\right)$. These changes affect the escape probability. If most extinction events of mutant lineages occur within a short time after mutation, we may be able to calculate $p\left(t\right)$ using $x\left(t\right)$ and $w\left(t\right)$ fixed at the values at which the mutant is produced. Under this approximation, the result is given as follows: ${p}_{SCA}\left(t\right)=1-\frac{h^{\prime }w\left(t\right)+\delta }{b^{\prime }x\left(t\right)}$, which we call "slow-change approximation."

In Appendix E, we explained this approximation in detail. Numerical analysis showed that the approximation was exact for the final stationary value $\overline{p }=p\left(\infty \right)$ and was relatively close for the initial value $p\left(0\right)$ when $p\left(0\right)>0.6.$ However, between these two situations, the formula deviates significantly from the exact value. This is plausible because the time change of $p\left(t\right)$ was zero for the stationary value and was small near $t=0$ but very fast between them. The parameter dependence shown in Fig. 6 indicates that the dependence of $p\left(0\right)$ on the nine parameters is consistent with the slow-change approximation. Further details are provided in Appendix E.

#### Time Required for $p\left(t\right)$ to Decline by 20%

Let ${\tau }_{p}$ be the time at which $p\left({\tau }_{p}\right)=0.8p\left(0\right)$ holds. This is the time for $p\left(t\right)$ to decrease by 20% of the initial level. We performed sensitivity analyses as explained in Appendix C.

#### Stationary Value $\overline{p }$

After the transient behavior, Eq. (1) converges to a stationary state, which is a stable equilibrium. The escape probability for a novel mutant produced at time $t$ also reaches its stationary value $\overline{p }=p\left(\infty \right)$.

**Fig. 7:**
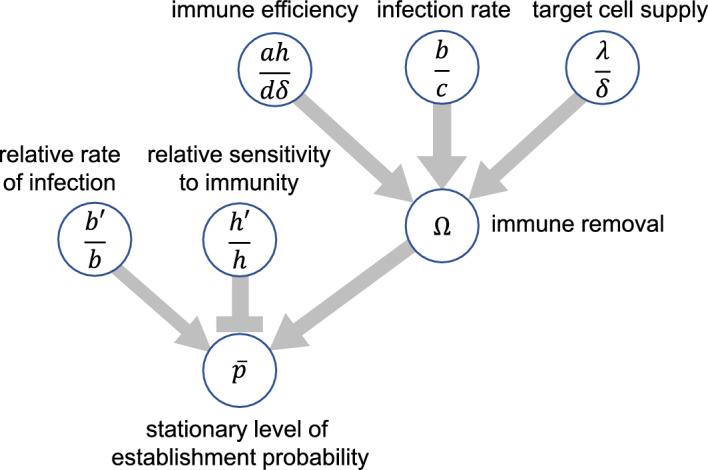
Scheme of the dependence of the stationary level of escape probability $\overline{p }$. It increases with ratio $b^{{\prime } } /b$ and decreases with ratio $h^{^{\prime }}/h$. In addition, it also depends on other parameters via $\Omega$, the importance of mortality by immune killing relative to random mortality. We assumed $0 < h^{{\prime } } < h$. Other parameters are the same as in Fig. 3. See Appendix A for derivation

According to calculation in Appendix A, the stationary value of escape (establishment) non-extinction probability $\overline{p }$ is affected by parameters as follows (summarized in Fig. 7 in Appendix A): $\overline{p }$ is determined by the relative rates of infection $b ^{\prime } /b$ and immune removal $h ^{\prime } /h$. In addition, $\overline{p }$ also depends on $\Omega =hw/\delta$, which is the immune removal relative to the natural mortality of cells infected by the wild-type strain. All other parameters affect $\overline{p }$ by modifying $\Omega$, which is the positive solution of the quadratic equation, $1=\left(\Omega +1\right)\left(\frac{\delta c}{\lambda b}\Omega +\frac{{\delta }^{2}d}{\lambda ah}\right)$. The importance of immunity compared to natural mortality $\Omega$ increases with the efficiency of the immune reaction to remove infected cells ($ah/d\delta$), the rate of infection of susceptible target cells ($b/c$), and the rate of supply of target cells ($\lambda /c$), as illustrated in Fig. 7 in Appendix A.

In the following two cases, we can obtain simple results:

Case 1. When the immune efficiency is the same between strains.

If $h ^{\prime } /h=1$ holds, the immune system activated by the wild-type strain operates on the mutants at the same strength (perfect cross-immunity). The stationary probability of escaping stochastic extinction is $\overline{p }=1-b ^{\prime } /b$ (see Appendix A for the derivation). This is the same as in our previous study (Hayashi et al. 2022), where the dynamics of the immune reaction were neglected. In the current paper, $p\left(t\right)$ depends on the dynamics of the immune reaction $w\left(t\right)$; however, the stationary value $\overline{p }$ is independent of the immune system.

Case 2. When the rate of infection of target cells is the same between strains.

By setting $b ^{\prime } /b=1$, we have $\overline{p }=\left(1-\frac{h ^{\prime } }{h}\right)\frac{\Omega }{\Omega +1}$, where $\frac{\Omega }{\Omega +1}$ indicates the fraction of mortality of infected cells killed by immune reactions, instead of natural mortality. The probability of escaping extinction is possible when the cross-immunity is weaker than perfect (i.e. $0<h ^{\prime } <h$).

Figure 6 indicates that $\overline{p }$ increases with $b ^{\prime } /b$ and decreases with $h ^{\prime } /h$. It also increases with $\lambda$, $a$, $b$, and $h$ but decreases with $c$, $d$, and $\delta$. These results are consistent with the parameter dependence of $\overline{p }$ (see Appendix C).

## Discussion

Pathogens threaten the lives of many organisms including human beings. Vertebrates have developed acquired immunity that prevents infection by the same pathogen twice. However, pathogens evolve by mutation to escape from the hosts’ immune responses. RNA viruses have a high mutation rate and exhibit rapid evolutionary adaptation, causing medical problems such as drug resistance and escape from immune surveillance ((Goulder et al. 1997; Althaus and Bonhoeffer 2005)). Hence, evaluating the emergence rate of mutants within a host is essential for disease control and medical treatment.

Grenfell et al. (2004) proposed “phylodynamics,” a methodology that predicts the epidemic dynamics of RNA viruses by considering viral evolution. In phylodynamics, the frequency and periodicity of pandemics were analyzed using epidemic models incorporating both the dynamics of host immunity and pathogen immune evasion. RNA viruses differ greatly in their epidemic dynamics and evolutionary patterns. These differences can be explained by the differences in the evolutionary potential of viruses to evade host immunity by mutation (antigenic drift) (Koelle et al. 2006; Lewis et al. 2008; Volz et al. 2013; Suchard et al. 2018; Saad-Roy et al. 2021). For example, measles with strong cross-immunity exhibits a highly synchronized and periodic infection. On the other hand, influenza A virus has immunity of intermediate strength, showing recurrent emergence of antigenically-different strains every year and an elongated molecular phylogeny of the virus (Grenfell et al. 2004).

The role of immune escape by mutant strains is also important in the within-host dynamics as well as in epidemiological dynamics. The immune system activated by the wild-type strain may remove cells infected by the mutant strain if they are similar in antigen type (cross-immunity). Many models have been studied to analyzed virus evolution under the influence of immune system (Nowak and May 2000). Most of these models of viral evolution within a host were deterministic. However, because of the stochasticity caused by the small initial number of cells, a large fraction of novel mutations go extinct even if they have a positive mean growth rate in the corresponding deterministic dynamics. This extinction may have a strong influence to the dynamics and evolution of the virus. For example, Sasaki and Haraguchi (Sasaki and Haraguchi 2000) highlighted that the loss of novel mutant viruses with a positive growth rate results in a prolonged existence of antigen drift within a host, rather than an explosion in the diversity of strains (Sasaki and Haraguchi 2000; Haraguchi and Sasaki 1997; Sasaki et al. 2012).

### Expected Number of Mutants that Escape Immune Surveillance

In our previous paper (Hayashi et al. 2022), we studied the temporal pattern of the probability of escape of a novel mutant virus within a target cell population of a host. However, we did not consider the effects of the host immune system. As a sequel, in the current paper, we studied the probability of establishing novel mutations when they experience cross-immunity that is weaker than the one attacking the wild-type strain. We again formalized the model as a continuous-time branching process when the growth rate and mortality of infected cells are given as functions of time. Unlike in Hayashi et al. (2022), we need to consider the time dependence of mortality caused by the immune system.

The rate of production of novel mutants that survive stochastic extinction is proportional to $f\left(t\right)=bx\left(t\right)y\left(t\right) \cdot p\left(t\right)$, the product of the number of infection events per unit time and the fraction of mutants that can escape the stochastic extinction risk. The potential emergence rate of novel mutants escaping extinction $f\left(t\right)$ had a sharp peak soon after infection with the wild-type virus (Fig. 3).

We observed that ${F}_{M}$, potential of novel mutations per patient produced during the initial phase of host infection, increased with cell-to-cell contact infection rate and decreased with susceptibility to cross-immunity, both being relative to the values of the wild-type strain ($b ^{\prime } /b$ and $h ^{\prime } /h$, respectively). If we fix these ratios, the expected number of novel mutations per patient attains its maximum in the case of immune activity of intermediate strength (see Fig. 4).

This result reminds us of how the rate of adaptation of RNA virus at the population level depends on host immunity. By studying epidemic dynamics incorporating immune reactions and viral evolution, Grenfell et al. (2004) observed that the evolutionary adaptation of RNA viruses to escape immunity occurs at the highest speed when the immune pressure is of intermediate strength. This is because very strong immune reactions quickly suppress the wild-type virus, making the number of mutations small, but very weak immune reaction does not favor mutants escaping immunity. Many subsequent theoretical studies incorporating the evolution of escape from immune surveillance have confirmed the usefulness of the phylodynamics concept (Koelle et al. 2006; Lewis et al. 2008; Volz et al. 2013; Suchard et al. 2018; Saad-Roy et al. 2021). Although it was proposed for epidemiological dynamics, a similar conclusion can be drawn for the viral dynamics within a single patient, as shown in the current study (Fig. 4).

### Viral Transmission Through Free Viral Particles

Many viruses proliferate within the host body by producing free viral particles that infect susceptible target cells, instead of cell-to-cell contact transmission. We examined the probability of escape of a mutant virus proliferating through free viral particles in the presence of immune responses activated by the wild-type strain by the following stochastic model similar to that in Hayashi et al. (2022): the proliferation event of an infected cell occurs at rate $r$ (i.e., an infected cell makes contact with an uninfected target cell and made the latter infected). At each event, the number of cells newly infected by viral particles produced from the infected cell follows a Poisson distribution with mean $\beta x$, proportional to the number of susceptible target cells. Simultaneously, the wild-type infected cell ruptures. The dynamics of $x$, $y$, and $w$, given by Eq. (1) hold if $b$ is replaced by $r\beta$ and $\delta$ is replaced by $\delta +r$ (see Appendix F for details). Hence, a positive stable equilibrium exists if $\lambda /c>\left(1/\beta \right)\left(1+\delta /r\right)$ holds (otherwise, no virus exists in the stable equilibrium). After a transient phase, $x\left(t\right)$ and $w\left(t\right)$ converge to the stationary levels. These are calculated by the dynamics given in Eq. (1).

The stochastic process of the abundance of a mutant strain is described by a branching process with time-dependent rates. We again considered $p\left(t\right)$, the escape probability of a mutant strain produced at time $t$. In Appendix F, we derived a differential equation for $p\left(t\right)$ and solve it numerically. We obtained $p\left(t\right)$ using a numerical integral with a sufficiently large $T$. The behavior of the model was similar to that of cell-to-cell contact transmission (see Appendix F).

### Stochastic Extinction of Advantageous Mutations

Stochastic extinction due to the small number of individuals plays an important role in many different fields of biology and medicine. For example, in evolutionary genetics theory, many advantageous mutations go extinct owing to stochasticity because they start from a small number. The fraction of novel mutants that can escape the extinction was first estimated by branching processes (Haldane 1927; Fisher 1930), which were extended to cover various aspects (Barton 1995; Johnson and Barton 2002; Oliveira and Campos 2004; Haccou et al. 2005; Peischl and Kirkpatrick 2012). A different line of mathematical formalism was based on diffusion processes, which handled the fixation of slightly deleterious mutations as well as the loss of advantageous mutations (Kimura 1957, 1962; Ohta 1972). In infectious diseases dynamics, branching process formalism provides tools for designing disease control strategies (Nishiura et al. 2012, 2017; Nakajo and Nishiura 2021). The stochastic extinction of small number of mutant cells is important in the study of the initiation and progression of cancer. The probability of cancer escaping drug or immune surveillance has been analyzed using stochastic processes (Iwasa et al. 2003, 2004, 2006; Michor et al. 2006; Foo et al. 2014).

The stochasticity plays important roles in many different aspects of viral dynamics and evolution within a host, which can be important themes of future theoretical studies. When a patient receives an antiviral drug treatment that suppresses the proliferation of the wild-type viral strain, the virus may mutate to a different antigen type that is resistant to the drug. Theoretical studies have been conducted on the emergence of drug-resistant mutations (Bonhoeffer and Nowak 1997; Ribeiro and Bonhoeffer 2000; Alexander and Bonhoeffer 2012). However, many of them were based on deterministic models. In our previous study (Hayashi et al. 2022), we discussed the temporal pattern of the escape (establishment) probability of a novel mutant in a patient who receives an antiviral drug. After drug administration starts, the wild-type strain stops proliferating and declines in abundance, and the target cell abundance recovers. Hence, the drug-resistant mutant enjoys an improved chance of escape. Because the mutant needs to be produced from the wild-type stain by mutation, the rate of production of drug-resistant mutants exhibits characteristic temporal patterns, with a sharp peak just before the drug administration (Hayashi et al. 2022). This scenario can be extended to cases in which the mutant enjoys the benefit of escaping immune suppression, in addition to drug resistance. This could be a topic for future theoretical study.

The cumulative viral load is defined as the time integral of the mutant virus abundance within a host. It has been used as an indicator of virulence in several studies (Iwasa et al. 2021; Marconi et al. 2011; Sempa et al. 2016, 2019; Kim et al. 2021; Iwanami et al. 2021). Iwasa et al. (2021) evaluated how the cumulative viral load depends on different processes. However, the analysis was performed based on deterministic dynamics. The effects of the stochastic extinction of the virus on the cumulative viral load in a patient could also be an important theme for future theoretical study.

## Figures and Tables

**Fig. 8:**
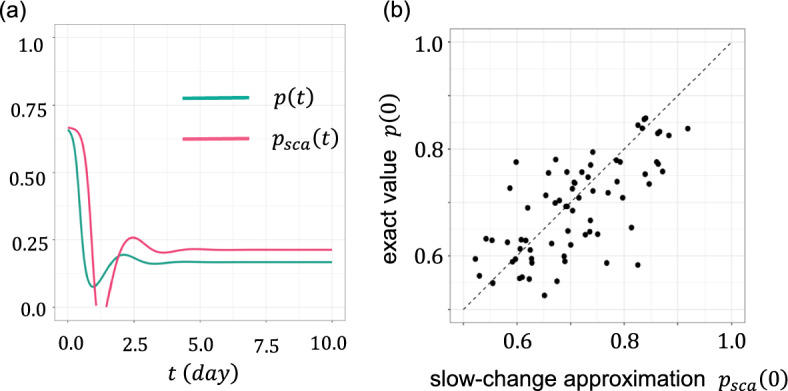
Slow-change approximation of $p\left(t\right)$. **a** Trajectory of escape probability $p\left(t\right)$ predicted by the slow-change approximation (refer to main text) and the exact value (Eq. (5)). Dots indicate the results of the different set of parameters. Note that the slow-change approximation was fairly accurate at $t=0$, and at the stationary value ($t=\infty$) but very different between these two limits. **b** ${p}_{SCA}\left(0\right)$ and exact value of $p\left(0\right)$, indicated in the vertical and horizontal axes, respectively. The diagonal line $y=x$ corresponds to cases when the approximate formula is exact. Parameters are the same as in Fig. 3. (See Appendix E for explanation)

## Data Availability

Data sharing not applicable to this article because no datasets were generated or analyzed during this study.

## Appendix A

### Equilibria of the Dynamics and the Stationary Value of Extinction Probability

Here, we consider the stable equilibrium of the dynamics for the number of susceptible target cells, number of cells infected by the wild-type virus, and immune reaction intensity, given by Eqs. (1a),(1b), and (1c), respectively. We set these equations to zero and obtain the following:

A.1a $$0=\lambda -cx-bxy$$

A.1b $$0=bxy-hwy-\delta y=y\left(bx-hw-\delta \right)$$

A.1c $$0=ay-dw$$

Let $\left(x,y,w\right)$ be the equilibrium of Eqs. (A1.a), (A1.b), and (A1.c). From Eq. (A.1b), at equilibrium, either [I] $y=0$ holds, or [II]$y>0$ and $bx-hw-\delta =0$ holds. At the equilibrium of type I, which contains no infected cells, we have $y=w=0$ and $x=\lambda /c$. This equilibrium always exists but may be locally stable or unstable. The stability of this equilibrium can be determined from $dy/dt=y\left(b\lambda /c-\delta \right)$, where we replace $x$ and $w$ with the equilibrium values. This equilibrium without infection is locally stable if $\lambda /c<\delta /b$. The equilibrium with infection does not exist; hence, the equilibrium is globally stable. In contrast, it is unstable if $\lambda /c>\delta /b$. Note that this inequality is the same as ${R}_{0}>1$, because the basic reproductive number is the product of the infection rate $b$, the number of susceptible target cells $(\lambda /c)$, and the mean longevity of the infected cell $(1/\delta )$.

When the equilibrium of type I is unstable, equilibrium of type II exists, where $y>0$ holds. We consider the cases in which the system has a positive steady state at which all three variables are positive (type II).

A.2a $$\lambda =cx+bxy$$

A.2b $$bx=hw+\delta$$

A.2c $$ay=dw$$

By examining the linearized dynamics of Eq. (1), we can show that the equilibrium of type (Nowak and May 2000), the solution of Eqs. (A.2), is always stable if a positive solution exists.

We can eliminate $y$ using $y=\left(d/a\right)w$, which can be derived from Eq. (A.2c). Then, from Eqs. (A.2a) and (A.2b), we obtain

A.3a $$\lambda =\frac{c}{b}bx+\frac{d}{a}bxw$$

A.3b $$bx=hw+\delta$$

By eliminating $bx$, we have

A.4 $$\lambda =\frac{c}{b}\left(hw+\delta \right)+\frac{d}{ah}\left(hw+\delta \right)hw$$

which becomes

A.5 $$1=\frac{\delta }{\lambda }\frac{c}{b}\left(\frac{hw}{\delta }+1\right)+\frac{{\delta }^{2}}{\lambda }\frac{d}{ah}\left(\frac{hw}{\delta }+1\right)\frac{hw}{\delta }$$

We set $\Omega =hw/\delta$, which is the ratio of mortality caused by the immune reaction to that caused by other mortality factors. This is a positive solution of

A.6 $$1=K\left(\Omega +1\right)+L\left(\Omega +1\right)\Omega$$

where $K=\left(\delta /\lambda \right)\left(c/b\right)$ and $L=\left(\delta /\lambda \right)\left(d\delta /ah\right)$. $K$ is larger when the number of target cells is limited. In contrast, $L$ is larger when the immune reaction is weak. Eq. (A.6) has a positive solution $\Omega$ when $K=\left(\delta /\lambda \right)\left(c/b\right)<1$, and has no positive solution when $K=\left(\delta /\lambda \right)\left(c/b\right)\ge 1$.

To obtain the positive equilibrium of the dynamics, we first obtain $\Omega$ from Eq. (A.6). Then we have $w=\left(\delta /h\right)\Omega$. Finally, we calculate $x=\left(hw+\delta \right)/b$ and $y=\left(d/a\right)w$.

### Probability of Escaping Extinction for a Mutant

The probability of escaping stochastic extinction emerging at time $t$ follows Eq. (4). The stable equilibrium of the dynamics when $x$ and $w$ are the values at stable equilibrium should satisfy the following equation:

A.7 $$0=\overline{p }\left(\left(1-\overline{p }\right)b ^{\prime } x-h ^{\prime } w-\delta \right)$$

From Eq. (A.7), the stationary value of $\overline{p }$ that is stable in the dynamics of Eq. (4) is given as.

A.8a $$\overline{p }=0, \,if\, b ^{\prime } x\le h ^{\prime } w+\delta.$$

A.8b $$\overline{p }=1-\frac{h ^{\prime } w+\delta }{b ^{\prime } x},\,if\, b ^{{\prime } } x>h ^{\prime } w+\delta.$$

Here, we consider how the escape probability $\overline{p }$ or extinction probability.

$1-\overline{p }$, depend on the parameters. In particular, we attempt to determine the impact of the relative magnitude of the rates of the focal mutant to those of the wild-type virus (such as $h ^{\prime } /h$ and $b ^{\prime } /b$). Eq. (A.8b) becomes

A.9 $$1-\overline{p }=\frac{\frac{h ^{\prime } }{h}hw+\delta }{\frac{b ^{\prime } }{b}bx}=\frac{\frac{h ^{\prime } }{h}\frac{hw}{\delta }+1}{\frac{b ^{\prime } }{b}\left(\frac{hw}{\delta }+1\right)}=\frac{\frac{h ^{\prime } }{h}\Omega +1}{\frac{b ^{\prime } }{b}\left(\Omega +1\right)}$$

### Parameter Dependence

The parameter dependence of the stationary value of extinction probability $1-\overline{p }$ depends on the relative vulnerability to the immune reaction ($h ^{\prime } /h$) and on the relative rate of infection to target cells ($b ^{\prime } /b$). It also depends on $\Omega =hw/\delta$, indicating the importance of mortality due to immune reactions relative to natural mortality.

As we consider the equilibrium with a positive abundance of viruses, the immune reaction is also positive, and $\Omega >0$ holds. From Eq. (A.9), we obtain

A.10a $$\frac{\partial }{\partial \frac{h ^{\prime } }{h}}\left(1-\overline{p }\right)=\frac{\Omega }{\frac{b ^{\prime } }{b}\left(\Omega +1\right)}>0$$

and

A.10b $$\frac{\partial }{\partial \frac{b ^{\prime } }{b}}\left(1-\overline{p }\right)=\left(-1\right)\frac{\frac{h ^{\prime } }{h}\Omega +1}{{\left(\frac{b ^{\prime } }{b}\right)}^{2}\left(\Omega +1\right)}<0$$

Then, the extinction probability increases with $h ^{\prime } /h$ and decreases with $b ^{\prime } /b$.

All other parameters affect the extinction probability through their effects on $\Omega .$ Considering the dependence of $\Omega$, as the positive solution of Eq. (A.6), we can interpret the parameters as follows: $\lambda /\delta$ is the rate of target cell supply relative to the decay rate, $b/c$ is the infection efficiency relative to the mortality of infected cells, and $ah/d\delta$ is the efficiency of immune reactions to the pathogen. Larger values of these quantities make $\Omega$ larger, which is the ratio of infected cell removal by immune reactions relative to the mortality by other processes. The greater impact of the immune reactions increases or decreases the stationary probability of escape (establishment) of the mutant virus. If the mutant can evade immune reactions more effectively than the wild-type strain, the greater impact of the immune reactions improves the escape probability.

A.11 $$\frac{\partial \overline{p}}{\partial\Omega }>0 \mathrm{because}\frac{h ^{\prime } }{h}<1$$

However, if the mutant suffered more immune reactions than the wild-type strain, the effect would be reversed. The impact of parameters on the stationary value of the escape probability $\overline{p }$ can be summarized by the scheme shown in Fig. 7.

### Simple Cases

We consider two simple cases:

Case 1. When the vulnerability to immune reaction is the same for the mutant and wild-type strains.

In this case, $h ^{\prime } /h=1$ holds. We have $1-\overline{p }=1/\left(b ^{\prime } /b\right)$, or $\overline{p }=1-b/b ^{\prime }$. This is the same as the condition when there is no immune reaction, derived in our previous study (Hayashi et al. 2022). The model includes the dynamics of immune reaction but it does not affect the stationary value of extinction probability for the mutant.

Case 2. When the rate of infection of the target cells is the same between the mutant and the wild-type strains.

Under this condition, $b ^{\prime } /b=1$ holds. We have the extinction probability $1-\overline{p }=\left(\left(h ^{\prime } /h\right)\Omega +1\right)/\left(\Omega +1\right)$, which is equal to 1 if $h ^{\prime } /h>1$, indicating that the extinction is certain ($\overline{p }=0$). If $h ^{\prime } /h<1$, the novel mutant stands a chance of surviving.

A.12 $$\overline{p }=\left(1-\frac{h ^{\prime } }{h}\right)\frac{\Omega }{\Omega +1}$$

The effect is proportional to $\Omega /\left(\Omega +1\right)=hw/\left(hw+\delta \right)$, which indicates the importance of removal by the immune system.

Figure 6 suggests that the stationary value $\overline{p }$ increased with $b ^{\prime } /b$ but decreased with $h ^{\prime } /h$. It also increased with $\lambda$, $b$, $a$, and $h$, but decreased with $\delta$, $c$, and $d$, which is consistent with Fig. 7.

## Appendix B

### Derivation of the Differential Equation for ${\varvec{p}}\left({\varvec{t}}\right)$

From Eq. (3) and the expression of ${p}_{2}\left(t\right)$ in the text, we have the following equation:

B.1 $$\begin{aligned}  p\left( t \right) & = b^{\prime } x\left( t \right)\Delta t\left( {1 - \left( {1 - p\left( {t + \Delta t} \right)} \right)^{2} } \right) + p\left( {t + \Delta t} \right) \\ &\quad + \left( { - b^{\prime } x\left( t \right)\Delta t - \left( {h^{\prime } w\left( t \right) + \delta } \right)\Delta t} \right)p\left( {t + \Delta t} \right) + o\left( {\Delta t} \right) \\ & = p\left( {t + \Delta t} \right) + \Delta tp\left( {t + \Delta t} \right)\left[ {b^{\prime } x\left( t \right)\left( {2 - p\left( {t + \Delta t} \right)} \right)  - b^{\prime } x\left( t \right) - h^{\prime } w\left( t \right) - \delta } \right] + o\left( {\Delta t} \right)\end{aligned} $$

Note that the difference between $p\left(t+\Delta t\right)$ and $p\left(t\right)$ is a small quantity of order $\Delta t$, we can rewrite the last expression of Eq. (B.1) as follows:

B.2 $$=p\left(t+\Delta t\right)+\Delta tp\left(t\right)\left[{b}^{{{\prime }}}x\left(t\right)\left(2-p\left(t\right)\right)-{b}^{{{\prime }}}x\left(t\right)-{h}^{{{\prime }}}w\left(t\right)-\delta \right]+o\left(\Delta t\right)$$

Hence, we have.

$$-\frac{\left(p\left(t+\Delta t\right)-p\left(t\right)\right)}{\Delta t}=p\left(t\right)\left[{b}^{^{\prime }}x\left(t\right)\left(1-p\left(t\right)\right)-{h}^{^{\prime }}w\left(t\right)-\delta \right]+\frac{o\left(\Delta t\right)}{\Delta t}$$

In the limit of $\Delta t\to 0$, we have

B.3 $$-\frac{dp}{dt}=p\left(t\right)\left[{b}^{{{\prime }}}x\left(t\right)\left(1-p\left(t\right)\right)-{b}^{{{\prime }}}x\left(t\right)-{h}^{{{\prime }}}w\left(t\right)-\delta \right]$$

which is the same as Eq. (4) in text.

### Derivation of the Integral Formula

Here, we consider the integration of the differential equation Eq. (4) in the main text. We consider the time derivative of the inverse of $p\left(t\right)$, as follows:

B.1 $$\frac{d}{{dt}}\left( {\frac{1}{{p\left( t \right)}}} \right) = - \frac{1}{{p\left( t \right)^{2} }}\frac{{dp}}{{dt}} = b^{\prime } x\left( t \right)\frac{1}{{p\left( t \right)}}\left( {1 - p\left( t \right)} \right) - \left( {h^{\prime } w\left( t \right) + \delta } \right)\frac{1}{{p\left( t \right)}} = - b^{\prime } x\left( t \right) + \left( {b^{\prime } x\left( t \right) - h^{\prime } w\left( t \right) - \delta } \right)\frac{1}{{p\left( t \right)}}$$

Because Eq. (B. 1) is a linear differential equation for a single variable $1/p\left(t\right)$ with time-dependent coefficients, we can obtain the time-dependent solution explicitly as follows:

B.2 $$\begin{aligned} & \frac{1}{p\left(t\right)}=\frac{1}{p\left(T\right)}exp\left[-{\int }_{t}^{T}\left({b}^{{{\prime }}}x\left(t ^{\prime } \right)-{h}^{{{\prime }}}w\left(t ^{\prime } \right)-\delta \right)d{t}^{{{\prime }}}\right]\\& +{\int }_{t}^{T}b ^{\prime } x\left(s\right)exp\left[-{\int }_{t}^{s}\left(b ^{\prime } x\left(t ^{\prime } \right)-h ^{\prime } w\left(t ^{\prime } \right)-\delta \right)dt ^{\prime } \right]ds\end{aligned}$$

We confirm that Eq. (B.2) satisfies differential equation (B.1) and the terminal condition at $t=T$. The mutant is advantageous because of either a faster infection rate or weaker immunity than those of the competing wild-type strain, and the mutant has a positive mean growth rate. Hence, when $T\to \infty$, we have ${\int }_{t}^{T}\left(b ^{\prime } x\left(t ^{\prime } \right)-h ^{\prime } w\left(t ^{\prime } \right)-\delta \right)dt ^{\prime } \to \infty$. As $T\to \infty$, Eq. (B.2) becomes:

B.3 $$\frac{1}{p\left(t\right)}={\int }_{t}^{\infty }b ^{\prime } x\left(s\right)exp\left[-{\int }_{t}^{s}\left(b ^{\prime } x\left(t ^{\prime } \right)-h ^{\prime } w\left(t ^{\prime } \right)-\delta \right)dt ^{\prime } \right]ds$$

which is rewritten as

B.4 $$p\left(t\right)=\frac{1}{{\int }_{t}^{\infty }b ^{\prime } x\left(s\right)exp\left[-{\int }_{t}^{s}\left(b ^{\prime } x\left(t ^{\prime } \right)-h ^{\prime } w\left(t ^{\prime } \right)-\delta \right)dt ^{\prime } \right]ds}$$

This formula is identical to Eq. (5) in the main text. Most of the numerical results for the model with the cell-to-cell contact transmission were generated using Eq. (B.4), or Eq. (5).

## Appendix C

We performed the sensitivity analysis of the model. We chose the following eight quantities to characterize the model's behavior: $p\left(0\right)$, ${\tau }_{p}$, $\overline{p }$, ${\tau }_{x}$, $\overline{x }$, ${\tau }_{f}$, $f\left({\tau }_{f}\right)$, and ${F}_{M}$. These values were calculated numerically from the trajectory of the dynamics given by Eq. (1) and Eq. (5). The model contains nine parameters: $\lambda$, $c$, $b$, $h$, $\delta$, $a$, $d$, $b ^{\prime } /b$, and $h ^{\prime } /h$.

Let $Q$ be one of the eight quantities. We evaluated $Q$ for the standard parameter set: for example, $a=10$, $b=2.5\times {10}^{-4}$, $c=1$, $d=5$, $\delta =5$, $\lambda =5\times {10}^{4}$, $h=5\times {10}^{-5}$, $b ^{\prime } /b=1.2$, and $h ^{\prime } /h=0.4$. These parameters are the same as the values adopted in drawing Fig. 3 and many other figures in this paper. We wanted to know the behavior of the model as a whole, rather than the case of a specific viral strain. How robust of the results of the sensitivity analysis for alternative choices of the standard parameter sets should be examined separately.

We also evaluated $Q$ for the case in which one parameter $k$ is shifted by a small magnitude $\Delta k$. The difference in $Q$ caused by this change is $\Delta Q=Q\left(k+\Delta k\right)-Q\left(k\right)$. We calculated $\frac{k}{Q}\frac{\Delta Q}{\Delta k}\approx \frac{\Delta logQ}{\Delta logk}$, and used it as a numerically calculated "elasticity," which is defined as ${e}_{k}=\frac{\partial logQ}{\partial logk}$. Note that the elasticity remains the same when we change the scale of the quantity (for example, it is independent of whether we measure length in terms of cm, mm, or $\mu$ m). Although defined in physics for materials, it has been widely adopted in economics, ecology, and virology (Iwasa et al. 2021; Acemoglu 2002; Kroon et al. 1986) as a useful measure of parameter sensitivity.

**Table 1:** Parameter sensitivity of eight quantities ($p\left(0\right)$, ${\tau }_{p}$, $\overline{p }$, ${\tau }_{x}$, $\overline{x }$, ${\tau }_{f}$, $f\left({\tau }_{f}\right)$, and ${F}_{M}$) characterizing the model's behavior on nine parameters ($\lambda$, $c$, $b$, $h$, $\delta$, $a$, $d$, $b^{{\prime } } /b$, and $h^{{\prime } } /h$) Elasticities were calculated using the method described in Appendix C. We generated many sets of parameters by randomly multiplying 2, 1, or 0.5 for each of nine parameters randomly to the one in Fig. 6, and calculated elasticities using the method described in Appendix C. Each number in the table shows the median of corresponding elasticity (from 58 to 100 values)

	$\lambda$	$c$	$b$	$h$	$\delta$	$a$	$d$	$\frac{{b^{{\prime } } }}{b}$	$\frac{{h^{{\prime } } }}{h}$
$p\left(0\right)$	$0.06$	$-0.03$	$0.56$	$0$.00	$-0.33$	$0$.00	$0$.00	$1.93$	$0.00$
${\tau }_{p}$	$0.65$	$-0.33$	$-1.26$	$0$.00	$-0.50$	$0.00$	$0$.00	$3.85$	$-0.09$
$\overline{p }$	$0.36$	$-0.11$	$0.14$	$0.10$	$-0.61$	$0.10$	$-0.11$	$3.07$	$-0.09$
${\tau }_{x}$	$0.19$	$-0.24$	$-0.46$	$0.00$	$0.00$	$0.00$	$0.00$	$0$	$0$
$\overline{x }$	$0.28$	$-0.11$	$-1.03$	$0.01$	$0.63$	$0.01$	$-0.02$	$0$	$0$
${\tau }_{f}$	$1.55$	$-1.20$	$1.93$	$0$.15	$-1.95$	$0.15$	$-09$	$4.59$	$-0.23$
$f\left({\tau }_{f}\right)$	$0.45$	$0.00$	$-1.43$	$0.00$	$0.00$	$0.00$	$0.00$	$0.78$	$0.00$
${F}_{M}$	$1.28$	$-0.57$	$0.34$	$0.15$	$-1.39$	$0.15$	$-0.09$	$4.32$	$-0.23$

Table  1 illustrates the elasticities of eight quantities characterizing the behavior of the model ($p\left(0\right)$, ${\tau }_{p}$, $\overline{p }$, ${\tau }_{x}$, $\overline{x }$, ${\tau }_{f}$, $f\left({\tau }_{f}\right)$, and ${F}_{M}$) on nine parameters ($\lambda$, $c$, $b$, $h$, $\delta$, $a$, $d$, $b ^{\prime } /b$, and $h ^{\prime } /h)$ at the standard parameter set. From these values, we can determine how $Q$ depends on parameter $k$. The exact values of elasticities may generally depend on the choice of the standard parameter set. We calculated the elasticities for many different choices of the standard parameter set. We produced 100 parameter sets by multiplying one of the three factors ($\times 2$, $\times 1$, $\times 0.5$) to each of the nine parameters. For those parameter sets that produced stable positive equilibria, we performed sensitivity analysis around the equilibrium. The median values of the sensitivities were shown in Table 1.

Many of the elasticities did not change sign. To indicate the robustness of the sensitivities, we classified the results to the following four situations: (Hayashi et al. 2022) the elasticity was greater than 0.01 for all cases; (Nowak and May 2000) the elasticity was less than -0.01 for all cases, (Iwasa et al. 2021) the elasticity was between -0.01 to 0.01 for all cases; and (Iwami et al. 2015) the elasticity showed mixed results. $p\left(0\right)$: elasticity was positive for $\lambda$, $b$, and $b ^{\prime } /b$; negative for $c$ and $\delta$; and is zero for $h$, $a$, and $d$. Elasticity for $h ^{\prime } /h$ was mostly zero but sometimes negative. ${\tau }_{p}$: elasticity was positive for $\lambda$ and $b ^{\prime } /b$; negative for $c$, $b$, $\delta$, and $h ^{\prime } /h$; zero for $h$, $a$, and $d$. $\overline{p }$: elasticity was positive for $\lambda$, $b$, $h$, $a$, and $b ^{\prime } /b$; negative for $c$, $\delta$, $d$, and $h ^{\prime } /h$. $\overline{x }$: elasticity was positive for $\lambda$, $h$, $\delta$, and $a$; negative for $c$, $b$, and $d$. ${\tau }_{f}$: elasticity was positive for $\lambda$, $b$, $h$, $a$, and $b ^{\prime } /b$; negative for $c$, $\delta$, $d$, and $h ^{\prime } /h$. $f\left({\tau }_{f}\right)$: elasticity was positive for $\lambda$, and $b ^{\prime } /b$; negative for $b$; zero for $d$. Elasticity was variable in sign for $c$ and $\delta$. It was mostly zero for $h$ and $a$, but sometimes positive; it was mostly zero for $h ^{\prime } /h$ but sometimes negative.

${F}_{M}$; elasticity was positive for $\lambda$, and $b ^{\prime } /b$; negative for $c$, $\delta$, and $h ^{\prime } /h$. Elasticity was variable in sign for $b$, $h$, $a$, and $d$,

Median of the elasticity in Table 1 gives an information on their magnitude.

## Appendix D

Numerical analyses of trajectories of the dynamics show that the rate of decline in $x$ is initially slow but accelerates overt time. The wild-type virus abundance $y$ and the strength of the immune reaction $w$ start from a small value (or zero) and increase in an accelerating manner. In this appendix, we discuss an approximate calculation near the onset of infection with the wild-type virus.

### Dynamics for Small ${\varvec{t}}$

Here we consider the behavior of the dynamics close to the time of initial infection by the original (wild-type) strain. For simplicity, we set the time of infection of the wild-type strain to 0 (${t}_{0}=0$), and consider the dynamics for small a small $t$.

The values of the three variables before the start of the infection are $x=\lambda /c$ and $y=w=0$. The initial values of the three variables are $x\left(0\right)=\lambda /c$, $y\left(0\right)=\varepsilon$, and $w\left(0\right)=0$, where $\varepsilon$ is a positive constant that is much smaller than $\lambda /c$.

We set $\widehat{x}\left(t\right)=x\left(t\right)-\lambda /c$. $\widehat{x}\left(t\right)$, $y\left(t\right)$, and $w\left(t\right)$ are quantities of the order $\varepsilon$. Equation (1) becomes

D.1a $$\frac{d\widehat{x}}{dt}=-c\widehat{x}-\frac{b\lambda }{c}y$$

D.1b $$\frac{dy}{dt}=\left(\frac{b\lambda }{c}-\delta \right)y$$

D.1c $$\frac{dw}{dt}=ay-dw$$

where we neglected small terms of higher order with respect to $\varepsilon$.

Differential equation (D.1b) with the initial condition $y\left(0\right)=\varepsilon$ gives

D.2a $$y\left(t\right)=\varepsilon \cdot exp\left[\eta t\right],$$

where we set $\eta =b\lambda /c-\delta$. We integrate Eq. (D.1a) with $\widehat{x}\left(0\right)=0$:

D.2b $$\widehat{x}\left(t\right)={\int }_{0}^{t}\left(-\frac{b\lambda }{c}\right)\varepsilon \cdot exp\left[\eta s\right]{e}^{-c\left(t-s\right)}ds=-\varepsilon \cdot \frac{b\lambda }{c} \frac{exp\left[\eta t\right]-exp\left[-ct\right]}{\eta +c}$$

Eq. (D.1c) together with the initial condition $w\left(0\right)=0$ gives

D.2c $$w\left(t\right)={\int }_{0}^{t}a\varepsilon \cdot exp\left[\eta s\right]{e}^{-d\left(t-s\right)}ds=\varepsilon \cdot \frac{a}{\eta +d}\left(exp\left[\eta t\right]-exp\left(-dt\right)\right)$$

From Eq. (D.2b), we have

D.2d $$x\left(t\right)=\frac{\lambda }{c}-\varepsilon \cdot \frac{b\lambda }{c} \frac{exp\left[\eta t\right]-exp\left[-ct\right]}{\eta +c}$$

By neglecting the term that decreases with time, we have further simplified formulas:

D.3a $${x}_{approx}\left(t\right)=\frac{\lambda }{c}\left(1-\varepsilon \cdot \frac{b}{\eta +c} exp\left[\eta t\right]\right)$$

D.3b $${y}_{approx}\left(t\right)=\varepsilon \cdot exp\left[\eta t\right]$$

D.3c $${w}_{approx}\left(t\right)=\varepsilon \cdot \frac{a}{\eta +d}exp\left[\eta t\right]$$

where $\eta =b\lambda /c-\delta$. Figure 5 illustrated these approximate formulas together with the exact solutions. We can see that Eq. (D.3a) overestimated the abundance of the target cells, and Eq. (D.3c) underestimated the immune intensity. However, the magnitude of the differences was rather small for a small $t$.

### Time Required for ${\varvec{x}}\left({\varvec{t}}\right)$ to Decline by 20%

Using the approximate formula (D.3a), we can derive the following estimate of ${\tau }_{x}$. From $x\left({\tau }_{x}\right)=0.8\frac{\lambda }{c}$, we have $0.2=\frac{\varepsilon b}{\eta +c} exp\left[\eta {\tau }_{x}\right]$, which becomes

D.4 $${\tau }_{x}=\frac{1}{\frac{\lambda b}{c}-\delta }log\left(\frac{0.2\left(\frac{\lambda b}{c}-\delta +c\right)}{\varepsilon b}\right)$$

## Appendix E

### Slow Change Approximation

The lineage starting from a mutant produced at time $t$ experiences temporal changes in the availability of target cells $x\left(t\right)$ and the intensity of immune reactions $w\left(t\right)$. The probability of escape depends on temporal changes in these rates. If most extinction events of mutant lineages occur within a short time after the mutant is produced, we can calculate $p\left(t\right)$ using $x\left(t\right)$ and $w\left(t\right)$ fixed to the values when the mutant is produced $t$. Under this approximation, the result is given as follows:

E.1 $${p}_{SCA}\left(t\right)=1-\frac{h^{\prime }w\left(t\right)+\delta }{b^{\prime }x\left(t\right)}$$

which we call "slow-change approximation."

In Fig. 8(a), we plotted the prediction of ${p}_{SCA}\left(t\right)$ together with the exact value of $p\left(t\right)$ given by the integral formula in Eq. (5). The approximation results in Eq. (E.1) must be exact for the stationary value $\overline{p }=p\left(\infty \right)$. In Fig. 5, the approximation is also close to the exact value for $p\left(0\right)$, the value at the onset of infection. However, it was very different in the transient phase, in which $p\left(t\right)$ changed rapidly. Note that the rate of change in $p\left(t\right)$ was small both at $t=0$ and for a very large $t$, where the slow-dynamics approximation was rather accurate. Because $x\left(0\right)=\uplambda /c$ and $w\left(0\right)=0$ hold before the infection of the wild-type strain, the slow-change approximation yields

E.2 $${p}_{SCA}\left(0\right)=1-\frac{c\delta }{\left({b}^{{{\prime }}}/b\right)\lambda b}$$

Eq. (E.2) increases with $b ^{\prime } /b$, $\lambda$, and $b$ and decreases with $c$ and $\delta$; however, it is independent of $h ^{\prime } /h$, $h$, $a$, or $d$. Figure 8 illustrates the exact value of $p\left(0\right)$ and the value given by Eq. (E.2), on the vertical and horizontal axes, respectively. The dots represent the results of the different sets of parameters (see caption to Fig. 8b). The slow-change approximation in Eq. (E.2) was positively correlated with the exact value when $p\left(0\right)>0.6$. However, they could be very different for $p\left(0\right)<0.6$ (not shown).

## Appendix F

Here, we study the case in which virus proliferation occurs through free viral particles.

In most cases, viruses proliferate within the host body by producing free viral particles that infect susceptible target cells, rather than through cell-to-cell contact transmission. In our previous paper (Hayashi et al. 2022), we studied the escape probability of a mutant virus with this mode of transmission. We can extend this analysis to a case that includes immune reactions activated by the wild-type strain.

For the virus proliferating through free viral particles, we consider the following model: the proliferation of an infected cell occurs at rate $r$ at random times. At each proliferation event, the number of cells newly infected by the viral particles produced from a single infected cell follows a Poisson distribution with mean $\beta x$, proportional to the number of susceptible target cells. Furthermore, the wild-type infected cell dies because it ruptures. These modifications change the dynamics of $x$, $y$, and $w$, as follows:

F.1a $$\frac{dx}{dt}=\lambda -cx-r\beta xy$$

F.1b $$\frac{dy}{dt}=r\left(\beta x-1\right)y-\left(hw+\delta \right)y$$

F.1c $$\frac{dw}{dt}=ay-dw$$

Eq. (F.1c) is the same as Eq. (1c).

Note that Eqs. (1a), (1b), and (1c) become Eqs. (F.1a), (F.1b), and (F1.c), respectively, if we replace $b$ by $r\beta$ and $\delta$ by $\delta +r$. This implies that the dynamics of Eq. (F.1) has a globally stable equilibrium with a positive abundance of infected cells if $\lambda /c>\left(1/\beta \right)\left(1+\delta /r\right)$ holds. If the opposite inequality holds, the dynamics have no equilibrium with a positive abundance of virus, and the one without the virus is globally stable. Below, we focus on the case in which $\lambda /c>\left(1/\beta \right)\left(1+\delta /r\right)$ holds.

### Differential Equation for the Escape Probability of the Mutant

The stochastic process of the abundance of a mutant strain is described by a branching process with a time-dependent rate constant. The number of target cells $x\left(t\right)$ is determined by the dynamics given in Eq. (F.1). We again consider $p\left(t\right)$, the escape probability of a mutant strain produced at time $t$.

Within a short time interval of $\Delta t$, a single cell ruptures with a probability $r\Delta t$. If this event occurs, the cell dies and releases numerous viral particles. If each susceptible target cell has a small probability to be infected by viral particles, the number of newly infected target cells follows a Poisson distribution with the mean proportional to the abundance of viral particles, which is assumed to be proportional to the number of infected cells. We assume that the number of cells that are newly infected by these viral particles follows a Poisson distribution with mean $\beta ^{\prime } x\left(t\right)$ and the infection efficiency greater than the one for the wild-type strain ($\beta ^{\prime } >\beta$). Consider a single cell at time $t$, which experiences the following transition until $t+\Delta t$. The cell may become $n-1$ cells with probability $r\Delta t{\left(\beta ^{\prime } x\left(t\right)\right)}^{n}\left(1/n!\right){e}^{-\beta ^{\prime } x\left(t\right)}$ ($n=0, 1, 2, 3,..$). It may be killed by random mortality with probability $\delta\Delta t$. Alternatively, the cell may remain unchanged, with a probability of $1-r\Delta t-\left(hw+\delta \right)\Delta t$. Here, we trace the number of target cells that are susceptible, infected by the wild-type strain, and infected by the mutant; however, we do not trace the number of free viral particles explicitly (Nowak and May 2000).

By considering a cell at time $t$ and occurring until time $t+\Delta t$, we obtain the following:

F.2 $$\begin{aligned} & p\left(t\right)=r\Delta t\left[{\sum }_{n=0}^{\infty }\frac{{\left({\beta }^{^{\prime }}x\left(t\right)\right)}^{n}}{n!}{e}^{-{\beta }^{^{\prime }}x\left(t\right)}{p}_{n}\left(t+\Delta t\right)\right]\\& +\left({h}^{^{\prime }}w\left(t\right)+\delta \right)\Delta t \cdot 0+\left(1-r\Delta t-\left(h^{\prime }w\left(t\right)+\delta \right)\Delta t\right)p\left(t+\Delta t\right)+o\left(\Delta t\right)\end{aligned}$$

where ${p}_{n}\left(t\right)$ is the probability for the lineage that starts from $n$ initial cells at time $t$ not to go extinct at time $T$. By assuming that the behavior of the lineages starting from different cells is independent of each other, we obtain the following equation: ${p}_{n}\left(t\right)=1-{\left(1-p\left(t\right)\right)}^{n}$ for $n=0, 1, 2, 3,..$, because the extinction of descendants starting from the initial $n$ cells is equivalent to the extinction of all the lineages. $o\left(\Delta t\right)$ is a term of higher order than $\Delta t$; hence, $o\left(\Delta t\right)/\Delta t\to 0$ when $\Delta t\to 0$.

As $\Delta t\to 0$, Eq. (F.2) becomes as follows.

$$-\frac{dp}{dt}=r\left[{\sum }_{n=0}^{\infty }\frac{{\left({\beta }^{^{\prime }}x\left(t\right)\right)}^{n}}{n!}{e}^{-{\beta }^{^{\prime }}x\left(t\right)}\left\{1-{\left(1-p\left(t\right)\right)}^{n}\right\}-p\left(t\right)\right] -\left(h^{\prime }w\left(t\right)+\delta \right)p\left(t\right)$$

which can be rewritten as

F.3 $$-\frac{dp}{dt}=r\left[1-{e}^{-{\beta }^{{{\prime }}}x\left(t\right)p\left(t\right)}-p\left(t\right)\right]-\left(h ^{\prime } w\left(t\right)+\delta \right)p\left(t\right)$$

We numerically integrated Eq. (F.3) from $t$ to $T$. The following terminal condition was used: $p\left(T\right)=1$. The target cell availability $x\left(t\right)$ is obtained from Eqs. (F.1a) and (F.1b), the dynamics of the wild-type strain and target cells, which are independent of the abundance of the mutant. However, in contrast to the quadratic equation in Eq. (4) and Appendix B, the nonlinear differential equation in Eq. (F.3) cannot be solved mathematically, and we do not have an explicit solution of $p\left(t\right)$ for a given $\left\{x\left(t\right)\right\}$, such as Eq. (B.4) or Eq. (5). Therefore, we must adopt the numerical integration of Eq. (F.3) with sufficiently large $T$.

### Stationary Level of Escape Probability

As explained in the main text, $x\left(t\right)$ starts from a high value, which is determined by the balance between supply and mortality of target cells. At time $t=0$, it decreases, and after damped oscillation, it converges to a stationary value $\overline{x }$. The intensity of the immune reaction $w\left(t\right)$ starts from 0, increases, and converges to a stationary value $\overline{w }$. We can obtain the stationary value of $p\left(t\right)=\overline{p }$ after $x\left(t\right)$ and $w\left(t\right)$ converge to $\overline{x }$ and $\overline{w }$, respectively, by setting $x\left(t\right)=\overline{x }$ and $w\left(t\right)=\overline{w }$ in Eq. (F.3). Thus, we have.

$$0=r\left[1-{e}^{-{\beta }^{^{\prime }}\overline{x }\overline{p} }-\overline{p }\right]-\left(h^{\prime }\overline{w }+\delta \right)\overline{p }$$

which is rewritten as

F.4 $$1-{e}^{-{\beta }^{{{\prime }}}\overline{x }\overline{p} }=\frac{{h}^{\prime } \overline{w} +\delta +r}{r}\overline{p }$$

We examined how $\overline{p }$ depended on the parameters of the model. Although there were some minor differences, parameter dependences were similar to those for cell-to-cell contact dynamics.
